# Quantum Phase Coherence in Mesoscopic Transport Devices with Two-Particle Interaction

**DOI:** 10.1038/srep12873

**Published:** 2015-08-10

**Authors:** Zhimei Wang, Xiaofang Guo, Haibin Xue, Naitao Xue, J.-Q. Liang

**Affiliations:** 1 Institute of Theoretical Physics, Shanxi University, Shanxi, Taiyuan 030006, China; 2 College of Physics and Optoelectrics, Taiyuan University of Technology, Taiyuan 030024, China

## Abstract

In this paper we demonstrate a new type of quantum phase coherence (QPC), which is generated by the two-body interaction. This conclusion is based on quantum master equation analysis for the full counting statistics of electron transport through two parallel quantum-dots with antiparallel magnetic fluxes in order to eliminate the Aharonov-Bohm interference of either single-particle or non-interacting two-particle wave functions. The interacting two-particle QPC is realized by the flux-dependent oscillation of the zero-frequency cumulants including the shot noise and skewness with a characteristic period. The accurately quantized peaks of cumulant spectrum may have technical applications to probe the two-body Coulomb interaction.

The QPC of conduction electrons has been an active research field for decades in the mesoscopic transport systems with the measuring of magnetic-flux-dependent current through an Aharonov-Bohm (AB) interferometer^1^. An AB interference experiment in the Coulomb blockade regime has been reported utilizing a bare AB ring with a quantum dot (QD) embedded within one arm of the ring^2^. The coherent properties provide new insight into the state of current transport in the dot. The two-particle interference has been investigated as a direct result of quantum exchange statistics in the absence of two-particle interaction^3^,^4^,^5^. The experimental realization of two-electron interference^5^ reproduces the original Hanbury Brown and Twiss experiments^6^,^7^ and has become a central study of multiple-particle wave functions. The QPC also has a great influence on the shot-noise properties^8^,^9^,^10^ from the viewpoint of transport electron-correlation. In fact, the quantum coherence of conduction-electron through different channels is caused physically by the phase accumulation of spatial motion from the electrode to QD.

The QPC with two-body Coulomb interaction (TBCI), however, has not yet been explored in a many-electron system. We in this report consider a double QD system weakly coupled to two metallic electrodes with two magnetic flux-lines of opposite directions as shown in Fig. 1. In the absence of the TBCI and inter-dot hopping, the single-particle as well as the two-particle AB phase interference^3^,^4^,^5^ does not exist at all since the total magnetic flux embraced by the electrode-dots-electrode loop is zero. We predict in this report a novel phenomenon of the QPC induced by the TBCI, which generates the wave-function overlap between two flux-lines and thus leads to the phase interference.

The new observation is demonstrated by the full counting statistics (FCS) for particle transport in mesoscopic systems. The FCS has attracted great attentions in recent years^8^,^9^,^10^,^11^,^12^,^13^,^14^,^15^,^16^,^17^,^18^,^19^,^20^,^21^,^22^,^23^,^24^,^25^,^26^,^27^,^28^,^29^,^30^,^31^ since the intrinsic properties of the mesoscopic system can be identified by the high-order cumulants of electron correlation besides the average transport-current itself^32^. It is believed that the high-order cumulants are useful to detect the zeros of generating function^33^ and the intrinsic multi-stability^34^. Moreover the fractional charge in a chiral Luttinger liquid^35^ is able to be extracted from the high-order cumulants and the Majorana bound-states in a nanowire as well. It is also well known that the high-order cumulants, e.g., the shot noise, the skewness, are more sensitive to the QPC effect than the average current in various types of QD systems^17^,^31^,^36^,^37^,^38^. Especially the fifteen-order^21^,^22^ and finite-frequency cumulants^25^ can be extracted from the real-time measurements in the electron transport through a semiconductor QD.

The main goal of this paper is to study the effect of TBCI on the QPC, which can be probed by the high-order cumulants of electron transport. A new type of QPC, which does not exist in the single- or two-electron transport in the absence of TBCI, is observed and well explained by the TBCI. We analyze the TBCI-induced QPC in the FCS based on the particle-number-resolved quantum master equation (QME), which provides a formulation to include the TBCI. We demonstrate how the QPC characterized by the flux-dependent oscillation of cumulants can be used to probe the TBCI.

We consider a double-QD weakly coupled to two metallic electrodes with two antiparallel magnetic flux-lines of Φ_1_ = *ζ*Φ_0_ and Φ_2_ = −Φ_1_, which are assumed to be perpendicular to the plane of QD-electrode seen from Fig. 1. Here, Φ_0_ = *ch*/*e* is the quantum unit of magnetic flux, and thus *ζ* is a dimensionless flux-number, which is a positive value when the magnetic field is pointed into the QD-electrode plan as that of the left-hand flux in Fig. 1. The reason for the arrangement of antiparallel magnetic fluxes is that the wave-function interference of single-particle or non-interacting two-particle can be avoided since the total flux embraced by the two-dot loop is zero. The TBCI induced QPC has not yet been reported to our knowledge. The Hamiltonian of the transport system is given by *H* = *H*_*dot*_ + *H*_*leads*_ + *H*_*T*_. Here the first term


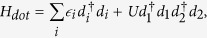


represents the QD Hamiltonian, where 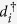 (*d*_*i*_) is the creation (annihilation) operator of an electron in the QD-*i* (*i* = 1, 2) and Œis the bare energy level of electron in the QD. *U* is the inter-dot Coulomb repulsion-strength between two electrons occupied respectively on two dots. In addition we assume the high intra-dot Coulomb interaction, so that the double-electron occupation in the same QD is prohibited. The electron transition between two dots, i.e. the inter-dot hopping, is neglected to avoid the AB interference around a single flux-line. In the considered case there exist total four occupation states of electrons on the two dots denoted respectively by 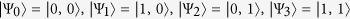 with corresponding energy-eigenvalues 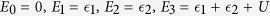. The term 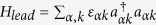 stands for the Hamiltonian of the two electrodes, with the energy dispersion *ε*_*αk*_, and 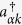 (*a*_*αk*_) being the creation (annihilation) operators of wave vector *k* in the *α*th electrode. The last term


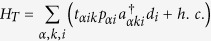


describes the tunneling coupling between the QD-*i* and the electrode-*α*. *p*_*αi*_ 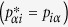 characterizes the flux-induced phases for electron hopping from the QD-*i* to the electrode-*α*, which are seen to be 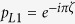, 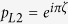, 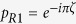, 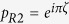 in the considered case, so that the total phase is zero from the electrode-*L* to *R* along one arm, for example, from the electrode-*L* through dot-1 to the electrode-*R*.

## Results

**Figure 2:**
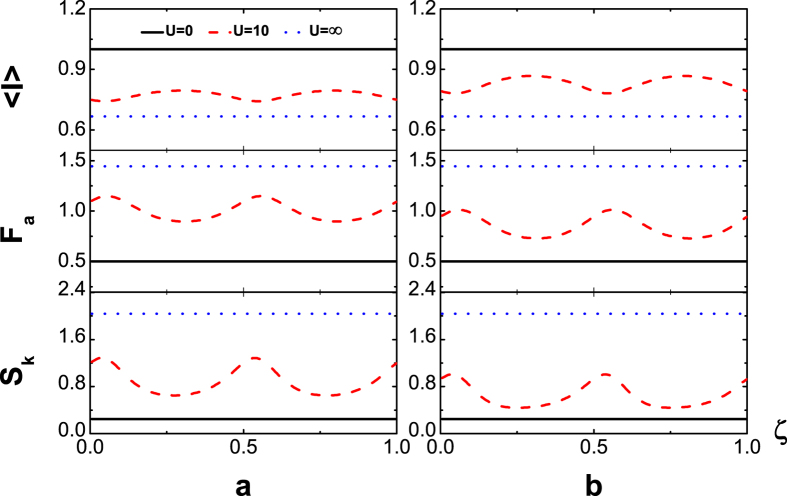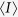 (upper panel), *F*_*a*_ (middle panel) and *S*_*k*_ (lower panel) versus flux-number *ζ* in the absence of inter-dot hopping with the symmetric dot-electrode coupling 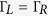 and 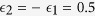, at temperatures *T* = 0 *K* 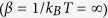 (**a**), and *T* = 8 *K* 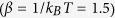 (**b**).

**Figure 3:**
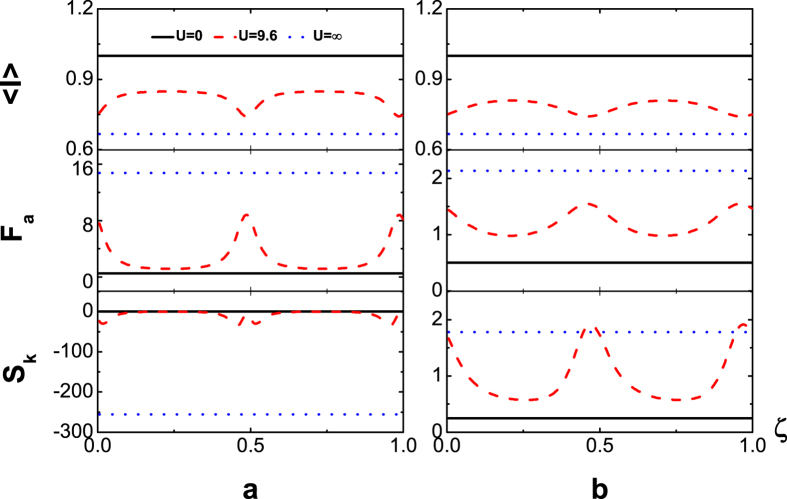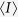 (upper panel), *F*_*a*_ (middle panel) and *S*_*k*_ (lower panel) versus flux-number *ζ* in the absence of inter-dot hopping with the symmetric dot-electrode coupling 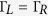 at zero temperatures when 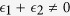, respectively for 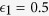, 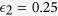 (**a**), 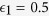, 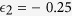 (**b**).

We now explore the QPC in the viewpoint of the FCS of electronic transport with a particular focus on the TBCI induced effect. The symmetrical bias-voltage 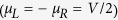 is assumed at the QD-electrode tunnel junctions with the energy unit *meV* in the typical experiment of transport^39^. In the numerical evaluations, the parameters are chosen as 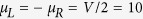. Based on the QME the electron number distribution *n*(*t*) can be evaluated in principle. We then calculate the cumulants of *n*(*t*), which, we will see, are more sensitive to the transport mechanism. The first-order cumulant defined as the average of electron number distribution 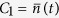 is related to average current obviously such that 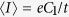. The second-order cumulants 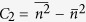 is the mean square deviation and is related to the zero-frequency shot noise defined by 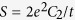. The third cumulant 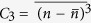 characterizes the skewness of the distribution. The over bar denotes the statistic average as usual that 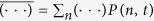. In general the normalized second-order cumulant 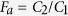, which is called the Fano factor, and the third cumulant 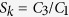 are used to describe the shot noise and the skewness respectively. The electron-number resolved statistics is called the super-Possonian for 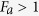 and sub-Possonian otherwise. We are particularly interested in the QPC, which depends on the magnetic flux number *ζ*. To this end we in Fig. 2 display the flux-number dependence of the average current 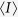 (upper panel), Fano factor *F*_*a*_ (middle panel) and skewness *S*_*k*_ (lower panel) at temperatures *T* = 0 *K* 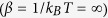 (a), and *T* = 8 *K* (*β* = 1.5) (b) with the symmetric dot-electrode coupling Γ_*L*_ = Γ_*R*_. In the absence of TBCI (black solid line) i.e. *U* = 0, the current curve does not vary with *ζ* as it should be, since the total flux embraced by the electron path-loop is zero no matter whether the dots are single- or double-occupied. On the other hand, for ultrastrong inter-dot Coulomb interaction such that *U* = ∞ (blue dotted line), which blocks the two-electron occupation states in the finite bias voltage, the average current does not depend on the flux again as the *U* = 0 case (black solid line). The flux-independence of the cumulants is explicitly verified from the analytic solution in the method for both *U* = 0 and *U* = ∞. When the Coulomb interaction is finite with the interaction strength *U* = 10 in Fig. 2 (red dashed line), the current oscillates with the flux number *ζ*. This phase interference is a result of the TBCI, which as a matter of fact provides the overlap of two-particle wave function between two flux lines. Most importantly, we observe a characteristic oscillation period 1/2 for the TBCI induced QPC, which is also robust against the thermal fluctuation at low temperature seen from Fig. 2(a,b). The *ζ*-dependence is derived rigorously in terms of the QME (see the detailed analysis in the section of method). Here we may present a plausible interpretation for the strict period from the two-electron scattering picture. For the two-particle wave functions transported from the electrode *L* to the two dots the Coulomb interaction in the semiclassical limit becomes 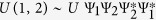, where 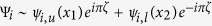 denotes the wave function of *i*th electron (*i* = 1, 2) transported from upper (*u*) and lower (*l*) paths to the dot-1, 2 respectively with *x*_1,2_ being the coordinates of dots. Notice that each dot can be occupied no more than one electron the Coulomb interaction term in the mean field approximation reduces to


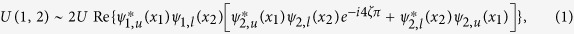


which varies with respect to *ζ* periodically and the period is precisely 1/2. For the electrons we may need the antisymmetric two-particle wave function, which does not affect the characteristic period-1/2. With the antisymmetric two-particle wave function


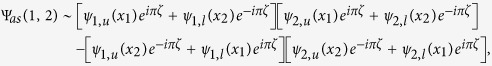


the two-electron Coulomb interaction becomes


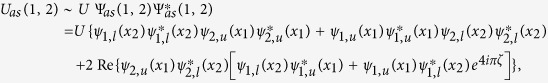


which leads again to the characteristic period-1/2 for the *ζ*–dependence. The two-electron transport probability depends also on the two-electron Fermi function 
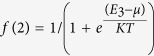
, which decreases with *U*. Thus the amplitude of current oscillation decreases with the increase of *U* and tends to zero when *U* → ∞, so that the *ζ*–dependence of current becomes a constant again (blue dotted line). The oscillations of Fano factor *F*_*a*_ and the skewness *S*_*k*_ are of the same period 1/2 and maximum values of *F*_*a*_ correspond to the minima of the current. This super-Possonian statistics of maximum values of *F*_*a*_ at zero temperature (a) is generated by the destructive interference of the TBCI induced QPC, which may be called the QPC blockade, namely, the effective bunching of the tunneling events. With the increase of temperature the Fano factor *F*_*a*_ and the skewness *S*_*k*_ decrease slightly seen from Fig. 2(b). The dot energy level, which is 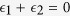 in Fig. 2, does not affect characteristic oscillation period of the *ζ*–dependence. The same plots, however, for 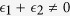 are displayed in Fig. 3, in which it is seen that the dot levels can greatly change the pattern shape but not the period.

**Figure 4:**
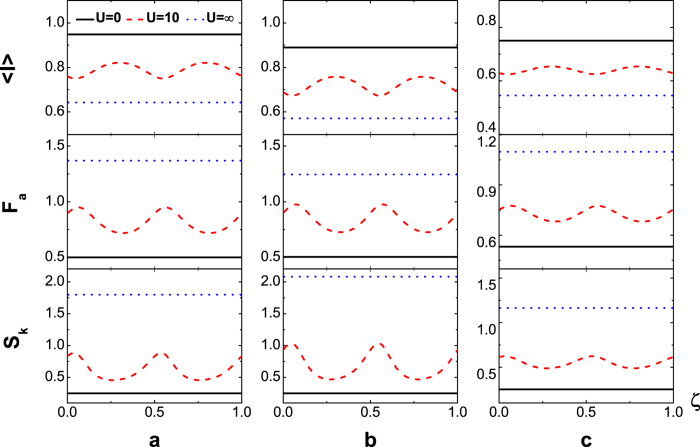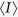 (upper panel), *F*_*a*_ (middle panel) and *S*_*k*_ (lower panel) versus flux-number *ζ* in the absence of inter-dot hopping at 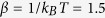 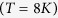 and 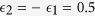, for asymmetric dot-electrode coupling 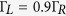 (**a**), 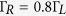 (**b**), 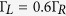 (**c**).

The characteristic oscillation period being 1/2 for finite Coulomb-interaction is also robust against the dynamic fluctuation, which may causes the asymmetry between electrode and dot couplings that 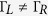. Figure 4 shows the *ζ*-dependence of 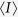 (upper panel), *F*_*a*_ (middle) and *S*_*k*_ (lower) at finite temperature *T* = 8 *K* for asymmetric dot-electrode coupling 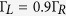 (a), 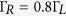 (b) and 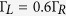 (c), respectively. Obviously, in these cases with the zero Coulomb-interaction (*U* = 0, black solid lines) and the ultrastrong interaction limit (*U* → ∞, blue dotted lines), 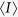, *F*_*a*_ and *S*_*k*_ are independent of the magnetic flux no matter whether the dot-electrode coupling is symmetric or not. However, in the presence of finite Coulomb interaction for *U* = 10 in Fig. 4, the evident oscillations are observed (red dashed lines). The oscillation period is 1/2 for the fixed bias voltage.

**Figure 5:**
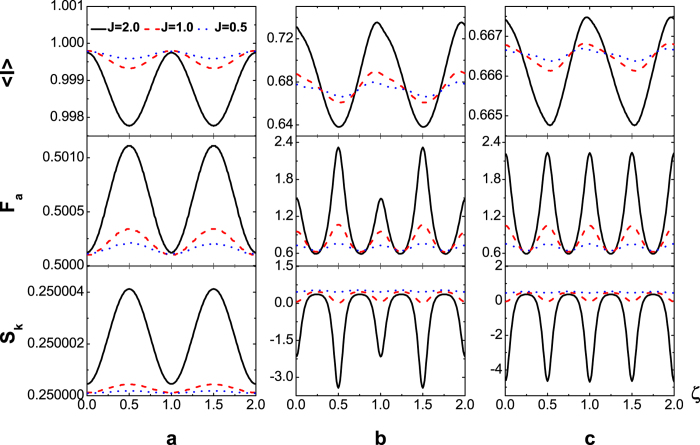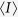 (upper panel), *F*_*a*_ (middle panel) and *S*_*k*_ (lower panel) versus flux-number *ζ* in the presence of inter-dot hopping for the Coulomb interaction strength *U* = 0 (**a**), *U* = 10 (**b**), and *U* = ∞ (**c**) with 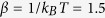 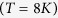, 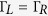 and 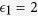, 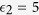.

We have demonstrated that in the absence of inter-dot hopping the QPC of the *ζ*-dependent oscillation is induced by the TBCI. To compare with the usual AB effect we now introduce the inter-dot hopping term 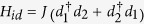. In this case (with the inter-dot hopping) the system thus becomes a AB interferometer of two series loops with each loop embracing a single flux-*ζ*. The energy eigenvalues and eigenstates of the coupled double dots are found as 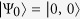, 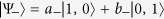, 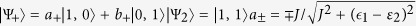, 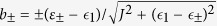, 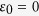, 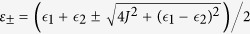, 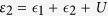. Figure 5 displays the 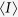 (upper panel), *F*_*a*_ (middle) and *S*_*k*_ (lower) as functions of flux number *ζ* with symmetric dot-electrode coupling 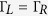 at finite temperature *T* = 8 *K* for the inter-dot Coulomb interactions *U* = 0 (a), *U* = 10 (b) and *U* = ∞ (c). The oscillation of current curve with respect to the flux-number *ζ* is the typical AB phase interference with period 1, which is the characteristic quantity of AB effect. The amplitudes of oscillation increase with the inter-dot coupling (*J* = 2 black solid line; *J* = 1 red dashed line; *J* = 0.5 blue dotted line). The average current decreases with the increase of inter-dot Coulomb interaction seen from Fig. 5(a–c) for 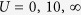 respectively due to the suppression of two-electron occupation probability. The Fano factor *F*_*a*_ is less than 1 for *U* = 0 (a) indicating sub-Possonian statistics and increases with *U* resulted from the Coulomb-interaction blockade (b) (c). For the finite interaction that *U* = 10 (b) the Coulomb interaction slightly changes the shape of interference pattern. The Fano factor *F*_*a*_ and skewness *S*_*k*_ are more sensitive to the phase interference. The *F*_*a*_ curves possess sharp peaks at half-integer values of *ζ* because of the QPC blockade while the *S*_*k*_ plots have corresponding sharp dips. The Coulomb interaction basically does not affect the oscillation period of average current with respect to *ζ*. However, additional peaks (dips) of *F*_*a*_ (*S*_*k*_) arise at the integer number of flux for the case of *U* = ∞ (c). This can be interpreted by the Coulomb blockade at the high current value (upper panel). The Coulomb-blockade induced peak-height of *F*_*a*_ increases with *U* and reaches the highest value at the limit case *U* = ∞. It should be noticed also an interesting phenomenon that the number statistics of transport electrons is super-Possonian at the peak position while sub-Possonian at dips for nonvanishing Coulomb interaction (b) (c). It may be worthwhile to remark that the *ζ*-dependence of transport current with the inter-dot hopping for *U* = 0 [Fig. 4(a)] is, as a matter of fact, the same as that in the non-interacting two-particle interference resulted from the quantum exchange statistics^3^,^4^,^5^ and becomes the usual single-particle AB interference at *U* = ∞.

## Conclusion and Discussion

In conclusion, the TBCI induced QPC has been explored for the first time according to the FCS of transport electrons. The observed flux-dependence of cumulants is different from the single- or non-interacting (i.e. *U* = 0) two-particle AB interference, since the total flux embraced by the two arms of electrode-dot-electrode paths is zero. Both the TBCI strength *U* and two-electron occupation probability are the necessary conditions to generate the magnetic-flux dependent QPC of the current 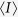, Fano factor *F*_*a*_ and skewness *S*_*k*_. In the strong interaction limit *U* → ∞ the phase interference does not appear as in the case of *U* = 0, since the two-electron occupation probability tends to zero reducing to the single-particle case. For the finite *U*, the period-1/2 oscillations are found in the absence of inter-dot hopping and can be interpreted by the TBCI, which actually provides the overlap probability of two-particle wave functions between two flux-lines leading to the nonvanishing phase coherence. This QPC is robust against dynamic asymmetry of coupling and also thermal fluctuation at low temperature. When the inter-dot hopping is introduced as a comparison the system becomes a AB interferometer with two series loops, for which the period of current oscillation is the typical value 1 of the AB interference and does not vary with the strength of Coulomb interaction *U*. However the higher order cumulant *F*_*a*_ (*S*_*k*_) displays additional peaks (dips) at the integer number of flux due to the Coulomb blockade and can serve as a probe to detect the TBCI.

## Methods

### Cumulant generating function matrix

The QD-electrode coupling is assumed to be sufficiently weak, such that the sequential tunneling is dominant. Therefore, the quantum transport can be well described by a QME of the reduced density matrix in the basis of eigenstates of the QDs. Regarding the Hamiltonian *H*_*T*_ as a perturbation, the second-order cumulant expansion leads to (under the Born and Markov approximations) the following particle-number-resolved QME for the reduced density matrix^19^,^40^,^41^


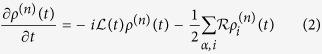


with


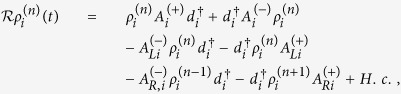


and


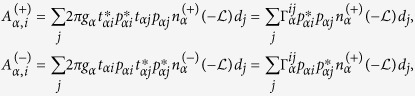


where 
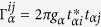
 denotes coupling strength relating the electrode-*α* and dots-*i*, *j*. 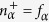, 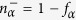 (*f*_*α*_ is the Fermi function of the electrode *α*), and *g*_*α*_ is the density of states in the electrode-*α*. The Liouvillian superoperator 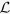 is defined as 
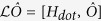
. 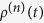 describes the reduced density matrix of the double-QD for *n* electrons tunneling through the junction up to time *t*. Throughout this paper, we set *ħ* = *e* = 1. In order to facilitate the calculation of FCS, we introduce the following cumulant generating function (CGF)^42^


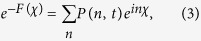


where *χ* is the counting field. The CGF *F*(*χ*) connects the particle-number-resolved density matrix 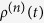 by 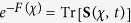, where the trace is over the eigenstates of double-QD and the column matrix 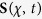 is defined by


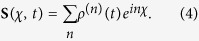


Evidently, one can obtain *e*^−*F*(*χ*)^ in terms of the column matrix 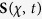. Since the particle-number-resolved density matrix Eq. (2) has the following form


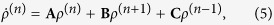


thus, 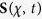 satisfies the equation


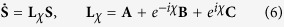


where **A**, **C** and **D** are three square matrices. We can obtain the explicit form of the matrix operator **L**_*χ*_ by processing a discrete Fourier transformation for the matrix element of Eq. (2).

The tunneling time through the QD system is much shorter than The counting time (i.e. the time of measurement) under the low frequency limit, thus *F*(*χ*) can be written as^17^,^18^,^19^,^42^,^43^,^44^,^45^


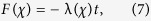


with λ(*χ*) being the eigenvalue of **L**_*χ*_ and tending to zero 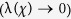 when *χ* → 0. Using the Eq. (7) one can obtain


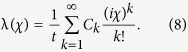


Plugging Eq. (8) into the Secular equation


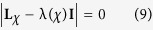


and extending the determinant in a series of (*iχ*)^*k*^, we can obtain the *k*th-order cumulant *C*_*k*_ by setting the coefficient of (*iχ*)^*k*^ being zero. The first three cumulants *C*_1_, *C*_2_ and *C*_3_ are obtained directly. We have seen that the CGF matrix **L**_*χ*_ is the key quantity to obtain the cumulant. The particle-number-resolved QME for the reduced density matrix (2) can be evaluated in the state basis 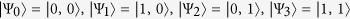. The result is explicitly written as


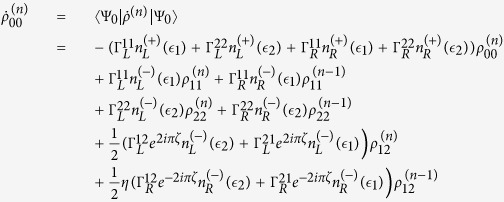



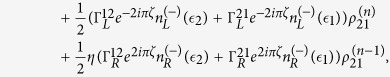



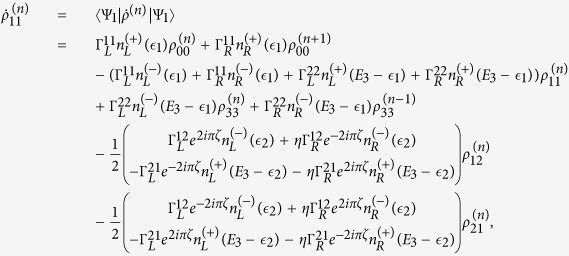



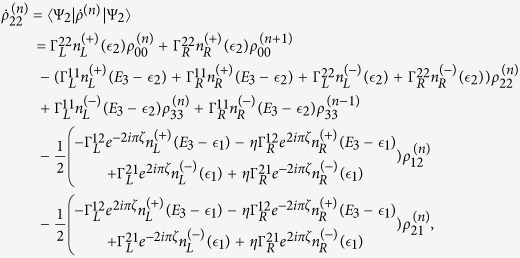



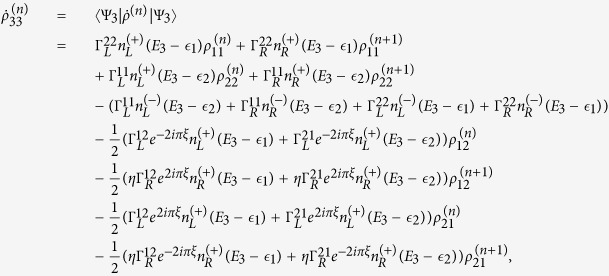



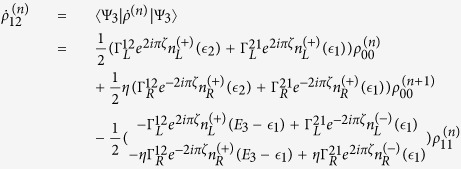



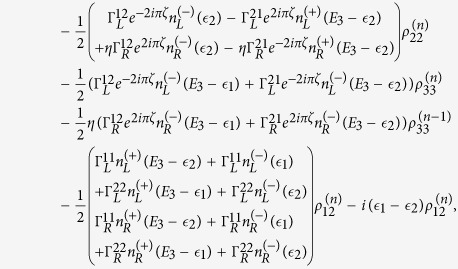



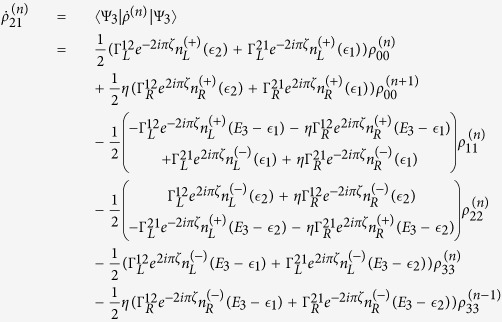



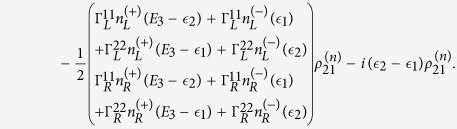


In this paper, we mainly discuss the quantum transport properties at extremely low temperature. In order to obtain an analytic formula we focus on the limiting case of zero temperature.

For the case of *U* = 0, the corresponding Fermi functions are seen to be 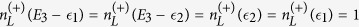 in the bias voltage region 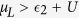, 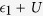, 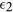, 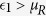 Thus, we obtain the CGF matrix **L**_*χ*_ as


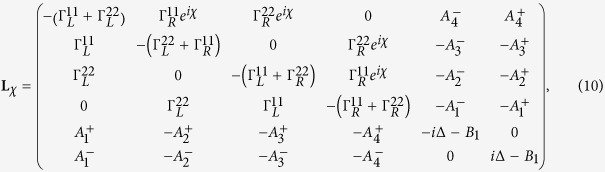


where


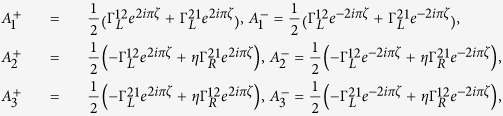



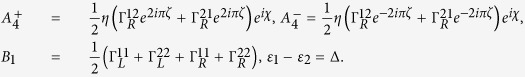


Performing the Taylor expansion of *e*^*iχ*^ the secular equation 
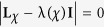
 reduces in the symmetric dot-electrode coupling 
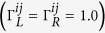
 case to a power series equation of *m*


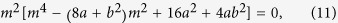


here 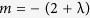, 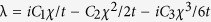, 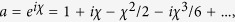 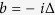. From Eq. (11) we can find the cumulants *C*_1_, *C*_2_, *C*_3_, which are independent of the magnetic flux number *ζ*. The *ζ*-independence is easy to understand since the net phase embraced by the wave functions is zero for both one and two particles transported from upper and lower arms of the loop in the absence of TBCI.

On the other hand when *U* = ∞ the corresponding Fermi functions become 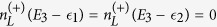, 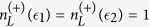 in the bias voltage region 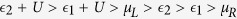, and thus the secular equation is seen to be


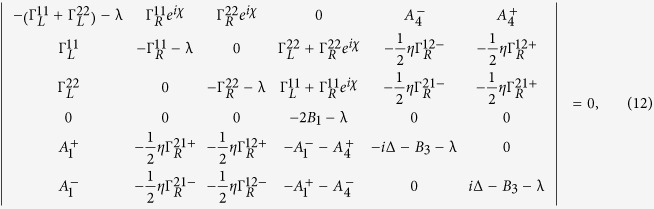


where 
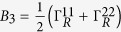
, 
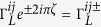
, 
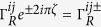
. At zero temperature the the secular equation reduces to a simple form


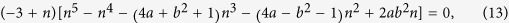


with 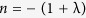. We again find that the cumulants *C*_1_, *C*_2_, *C*_3_ are *ζ*-independent. The reason for the independence is also obvious since the double occupation of QDs is zero at finite bias voltage for *U* = ∞ and the system reduces to a single-particle interferometer with zero total flux.

However for the finite TBCI with *U* = 10 the corresponding Fermi functions become 
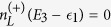
, 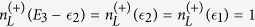 in the bias region 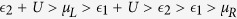. The matrix operator is found as


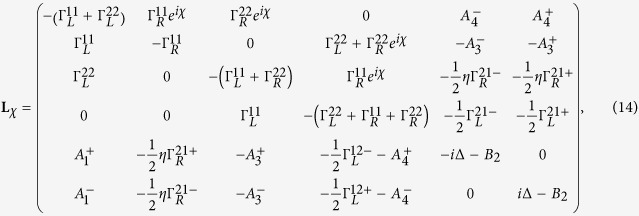


with 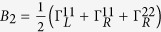. The secular equation does not have a simple form of power series and the cumulants *C*_1_, *C*_2_, *C*_3_ are *ζ*-dependent resulted from the dot-electrode couplings 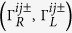, which are *ζ*-dependent including the phases 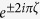. The TBCI provides the overlap of two-particle wave functions between two fluxes leading to the *ζ*-dependent phase coherence.

## Additional Information

**How to cite this article**: Wang, Z. *et al.* Quantum Phase Coherence in Mesoscopic Transport Devices with Two-Particle Interaction. *Sci. Rep.* **5**, 12873; doi: 10.1038/srep12873 (2015).

## Figures and Tables

**Figure 1:**
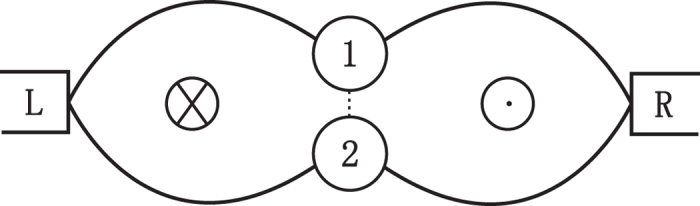
Schematic diagram of a double quantum-dot device with two antiparallel fluxes and tunable inter-dot Coulomb interaction.

## References

[b1] Holleitner A.-W., Decker C.-R., Qin H., Eberl K. & Blick R.-H. Coherent Coupling of Two Quantum Dots Embedded in an Aharonov-Bohm Interferometer. Phys. Rev. Lett. 87, 256802 (2001). 11736594 10.1103/PhysRevLett.87.256802

[b2] Yacoby A., Heiblum M., Mahalu D. & Shtrikman H. Coherence and Phase Sensitive Measurements in a Quantum Dot. Phys. Rev. Lett. 74, 4047 (1995). 10058399 10.1103/PhysRevLett.74.4047

[b3] Yurke B. & Stoler D. Bell’s-inequality experiments using independent-particle sources. Phys. Rev. A 46, 2229 (1992). 9908377 10.1103/physreva.46.2229

[b4] Samuelsson P., Sukhorukov E. V. & Buttiker M. Two-particle Aharonov-Bohm effect and entanglement in the electronic Hanbury Brown-Twiss setup. Phys. Rev. Lett. 92, 026805 (2004). 14753955 10.1103/PhysRevLett.92.026805

[b5] Neder I. *et al.* Interference between two indistinguishable electrons from independent sources. Nature 448, 333 (2007). 17637665 10.1038/nature05955

[b6] Hanbury Brown R. & Twiss R. Q. Correlation between photons in two coherent beams of light. Nature 177, 27 (1956).

[b7] Hanbury Brown R. & Twiss R. Q. A new type of interferometer for use in radio astronomy. Phil. Mag. 45, 663 (1954).

[b8] Dong B., Lei X.-L. & Horing N. J. M. Finite-frequency current (shot) noise in coherent resonant tunneling through a coupled-quantum-dot interferometer. J. Appl. Phys. 104, 033532 (2008).

[b9] Qin L. & Guo Y. Tunable shot noise in parallel-coupled double quantum dotsąŕsystem. J. Phys.: Condens. Matter 20, 365206 ( 2008).

[b10] Urban D. & König J. Tunable dynamical channel blockade in double-dot Aharonov-Bohm interferometers. Phys. Rev. B 79, 165319 (2009).

[b11] Lu W., Ji Z., Pfeier L., West K.-W. & Rimberg A.-J. Real-time detection of electron tunnelling in a quantum dot. Nature 423, 422 (2003). 12761544 10.1038/nature01642

[b12] Fujisawa T., Hayashi T., Hirayama Y., Cheong H.-D. & Jeong Y.-H. Electron counting of single-electron tunneling current. Appl. Phys. Lett. 84, 2343 (2004).

[b13] Gustavsson S. *et al.* Counting statistics of single-electron transport in a quantum dot. Phys. Rev. Lett. 96, 076605 (2006). 16606120 10.1103/PhysRevLett.96.076605

[b14] Fujisawa T., Hayashi T., Tomita R. & Hirayama Y. Bidirectional counting of single electrons. Science 312, 1634 (2006). 16778051 10.1126/science.1126788

[b15] Djurica I., Dong B. & Cui H.-L. Super-Poissonian shot noise in the resonant tunneling due to coupling with a localized level. Appl. Phys. Lett. 87, 032105 (2005).

[b16] Aghassi J., Thielmann A., Hettler M.-H. & Schön G. Shot noise in transport through two coherent strongly coupled quantum dots. Phys. Rev. B 73, 195323 (2006).

[b17] Kießlich G., Samuelsson P., Simovič B., Wacker A. & Schöll E. Counting statistics and decoherence in coupled quantum dots. Phys. Rev. B 73, 033312 (2006).

[b18] Groth C.-W., Michaelis B. & Beenakker C. W. J. Counting statistics of coherent population trapping in quantum dots. Phys. Rev. B 74, 125315 (2006).

[b19] Wang S.-K., Jiao H.-J., Li F., Li X.-Q. & Yan Y.-J. Full counting statistics of transport through two-channel Coulomb blockade systems. Phys. Rev. B 76, 125416 (2007).

[b20] Lindebaum S., Urban D. & König J. Spin-induced charge correlations in transport through interacting quantum dots with ferromagnetic leads. Phys. Rev. B 79, 245303 (2009).

[b21] Flindt C. *et al.* Universal oscillations in counting statistics. Proc. Natl. Acad. Sci. USA 106, 10116 (2009). 19515823 10.1073/pnas.0901002106 PMC2700917

[b22] Fricke C., Hohls F., Sethubalasubramanian N., Fricke L. & Haug R.-J. High-order cumulants in the counting statistics of asymmetric quantum dots. Appl. Phys. Lett. 96, 202103 (2010).

[b23] Luo J.-Y., Shen Y., He X.-L., Li X.-Q. & Yan Y.-J. Full counting statistics of renormalized dynamics in open quantum transport system. Phys. Lett. A 376, 59 (2011).

[b24] Jin J.-S., Li X.-Q., Luo M. & Yan Y.-J. Non-Markovian shot noise spectrum of quantum transport through quantum dots. J. Appl. Phys. 109, 053704 (2011).

[b25] Ubbelohde N., Fricke C., Flindt C., Hohls F. & Haug R.-J. Measurement of finite-frequency current statistics in a single-electron transistor. Nat. Comms. 3, 612 ( 2012). 10.1038/ncomms1620 PMC3272564 22215087

[b26] Choi T., Ihn T., Schön S. & Ensslin K. Counting statistics in an InAs nanowire quantum dot with a vertically coupled charge detector. Appl. Phys. Lett. 100, 072110 (2012).

[b27] Xue H.-B., Zhang Z.-X. & Fei H.-M. Tunable super-Poissonian noise and negative differential conductance in two coherent strongly coupled quantum dots. Eur. Phys. J. B 85, 336 (2012).

[b28] Komijani Y. *et al.* Counting statistics of hole transfer in a p-type GaAs quantum dot with dense excitation spectrum. Phys. Rev. B 88, 035417 (2013).

[b29] Fricke L. *et al.* Counting Statistics for Electron Capture in a Dynamic Quantum Dot. Phys. Rev. Lett. 110, 126803 (2013). 25166833 10.1103/PhysRevLett.110.126803

[b30] Zhang H.-W., Xue H.-B. & Nie Y.-H. Full counting statistics of a quantum dot doped with a single magnetic impurity. AIP Advances 3, 102116 ( 2013).

[b31] Xue H.-B. Full counting statistics as a probe of quantum coherence in a side-coupled double quantum dot system. Annals of Physics 339, 208 (2013).

[b32] Blanter Y.-M. & Bütiker M. Shot noise in mesoscopic conductors. Phys. Rep. 336, 1 ( 2000).

[b33] Kambly D., Flindt C. & Büttiker M. Factorial cumulants reveal interactions in counting statistics. Phys. Rev. B 83, 075432 (2011).

[b34] Schaller G., Kießlich G. & Brandes T. Counting statistics in multistable systems. Phys. Rev. B 81, 205305 (2010).

[b35] Komnik A. & Saleur H. Full Counting Statistics of Chiral Luttinger Liquids with Impurities. Phys. Rev. Lett. 96, 216406 (2006). 16803262 10.1103/PhysRevLett.96.216406

[b36] Welack S., Esposito M., Harbola U. & Mukamel S. Interference effects in the counting statistics of electron transfers through a double quantum dot. Phys. Rev. B 77, 195315 (2008).

[b37] Fang T.-F., Wang S.-J. & Zuo W. Flux-dependent shot noise through an Aharonov-Bohm interferometer with an embedded quantum dot. Phys. Rev. B 76, 205312 (2007).

[b38] Fang T.-F., Zuo W. & Chen J.-Y. Fano effect on shot noise through a Kondo-correlated quantum dot. Phys. Rev. B 77, 125136 (2008).

[b39] Elzerman J.-M. *et al.* Single-shot read-out of an individual electron spin in a quantum dot. Nature 430, 431 (2004). 15269762 10.1038/nature02693

[b40] Li X.-Q., Cui P. & Yan Y.-J. Spontaneous Relaxation of a Charge Qubit under Electrical Measurement. Phys. Rev. Lett. 94, 066803 (2005). 15783765 10.1103/PhysRevLett.94.066803

[b41] Li X.-Q., Luo J., Yang Y.-G., Cui P. & Yan Y.-J. Quantum master-equation approach to quantum transport through mesoscopic systems. Phys. Rev. B 71, 205304 (2005).

[b42] Bagrets D.-A. & Nazarov Yu.-V. Full counting statistics of charge transfer in Coulomb blockade systems. Phys. Rev. B 67, 085316 (2003).

[b43] Flindt C., Novotný T. & Jauho A.-P. Full counting statistics of nano-electromechanical systems. Europhys. Lett. 69, 475 (2005).

[b44] Flindt C., Novotný T., Braggio A., Sassetti M. & Jauho A.-P. Counting Statistics of Non-Markovian Quantum Stochastic Processes. Phys. Rev. Lett. 100, 150601 (2008). 18518090 10.1103/PhysRevLett.100.150601

[b45] Flindt C., Novotný T., Braggio A. & Jauho A.-P. Counting statistics of transport through Coulomb blockade nanostructures: High-order cumulants and non-Markovian effects. Phys. Rev. B 82, 155407 (2010).

